# Hidden blood loss of minimally invasive hybrid lumbar interbody fusion: an analysis of influencing factors

**DOI:** 10.1186/s12891-022-06079-x

**Published:** 2022-12-15

**Authors:** Zhong Dai, Da Peng Feng, Kang Long Wu, Jie Yang Zhu, Zheng Wei Li

**Affiliations:** The Second Affliated Hospital of Dalian Medical University, 467# ZhongShan Road, Dalian, Liaoning Province People’s Republic of China

**Keywords:** Lumbar interbody fusion, Hidden blood loss, Risk factors

## Abstract

**Background:**

Lumbar interbody fusion(LIF) is the leading way to treat Lumbar Degenerative Diseases(LDD). At present, there is a lack of research on the influencing factors of hidden blood loss in minimally invasive hybrid lumbar interbody fusion. This study comprehensively explores the definite factors affecting the hidden blood loss in minimally invasive hybrid lumbar interbody fusion.

**Materials and methods:**

One hundred patients with Lumbar degenerative diseases who underwent minimally invasive hybrid lumbar interbody fusion in our center were included. Demographics, laboratory data, surgical data, and radiographic data were collected. The Gross equation and Sehat equation were used to calculate the estimated value of hidden blood loss. Multi-factor linear regression analysis was used to determine the influencing factors of hidden blood loss.

**Result:**

We reviewed and collected 100 patients who underwent minimally invasive hybrid approach, mean age 65 ± 10 years, male: female 37:63; 17 patients of diabetes and 83 patients of non-diabetes; Total blood loss was 645.59 ± 376.37 ml, hidden blood loss was 421.39 ± 337.45 ml, the hidden blood loss percentage was 57 ± 26%. Results from the multi-factorial linear regression model: Diabetes (*p* < 0.05), hypertension (p < 0.05), psoas thickness (*p* < 0.05) and dorsal extensor group thickness (p < 0.05) were potential risk factors for postoperative hidden blood loss.

**Conclusion:**

Although minimally invasive hybrid approach is minimally invasive surgery, there is still a significant amount of hidden blood loss. There is a greater risk of blood loss in diabetes, hypertension and preoperative MRI assessment of thickness of the psoas, thickness of the dorsal extensor group.

## Background

Lumbar Degenerative Diseases (LDD) are the most frequent diseases in spinal surgery, and the frequency of lumbar degenerative diseases is increasing due to societal development and changes in lifestyle [1]. Intervertebral disc degeneration is closely linked to the production of inflammatory factors by the body. For patients with chronic low back pain, conservative treatment may be effective because physiotherapy is associated with anti-inflammatory and regenerative effects [2]. Conversely, surgical treatment is necessary for patients who have failed conservative treatment, or who have significant radicular symptoms. Anterior lumbar interbody fusion (ALIF) and oblique lateral lumbar interbody fusion (OLIF) are two well-established minimally invasive spinal fusion techniques for treating degenerative lumbar spine diseases [3, 4]. The advancement in surgical and perioperative treatment has resulted in a significant decrease in intraoperative blood loss. However, hidden blood loss after wound closure in spinal surgery is often higher than intraoperative bleeding [5]. Multi-segment posterior lumbar decompression fusion with higher hidden blood loss than visible blood loss [6]. Mima reported that HBL for XLIF was eight times greater than intraoperative bleeding [7]. It also explains why the patient’s postoperative condition is frequently incompatible with intraoperative hemorrhage, which could be related to the patient’s hidden blood loss following surgery [8]. Postoperative blood transfusions are always indispensable if the patient’s HBL is > 850 ml [9].

Previous research has linked minimally invasive spinal methods to considerable hidden blood loss, and there are numerous possible risk factors for hidden blood loss [10–13]. A new attempt to integrate various treatments, including ALIF, OLIF, and percutaneous pedicle screw fixation (PPSF), is the minimally invasive hybrid approach. However, there is still a scarcity of studies on the factors that influence Minimally invasive hybrid approach hidden blood loss. As a result, the objective of our study was to examine the quantity of hidden blood loss and associated risk factors during a minimally invasive hybrid approach, as well as to establish a reasonable blood management strategy.

## Data and methods

### Ethical review

The clinical data of LDD patients who underwent Minimally invasive hybird approach in the Second Affiliated Hospital of Dalian Medical University from November 2020 to April 2022 were retrospectively collected and analyzed. This study was approved by the Ethics Review Committee of the Second Affiliated Hospital of Dalian Medical University and obtained the unique identification number of research registration (the research registration number is 2022055). Each patient signed a written informed consent form.

### Inclusion and exclusion criteria

The inclusion criteria include: ① Patients with clinically diagnosed degenerative lumbar disease. ② Multi-segment LDD (L2-S1 Segmental continuity, Essential Including L5-S1 level) ③Minimally invasive hybird approach was performed.

The exclusion criteria include: ①Lack of information including demographics, Laboratory data, surgical data, radiographic data. ②Patients with severe hematological disorders and severe cardiovascular disease. ③with lumbar infections or tumors. ④with previous lumbar surgery. ⑤with autologous and allogeneic blood transfusion.

A total of 124 patients were performed by Minimally invasive hybird approach during the study period. 100 patients were enrolled after applying the inclusion and exclusion criteria. Eight patients with lumbar infections or tumors,6 patients with previous lumbar surgery,5 patients with autologous and allogeneic blood transfusion,3 patients with Incomplete information,2 patients with coagulopathy were excluded.

### Operation method

All the operations were performed by the same team in our hospital. All patients were given general anesthesia and were performed by Minimally invasive hybird approach.

Surgery sequence: ALIF → OLIF → PPSF. In our experience, ALIF procedure is first performed to open the L5-S1 gap, especially in patients with high iliac, to facilitate the L4–5 segment surgery.

①ALIF: The anterior approach was midline of L5-S1 level, and the cartilage of endplate was removed by conventional methods after discectomy. The endplate was prepared with a straight curette. For narrow disc space, the distraction was performed using a parallel distractor. Proper implant size was determined using the trial implants under fluoroscopy. The cage filled with the bone grafting material was inserted into the intervertebral space using fluoroscopy. Once the position of the cage was optimal, anterior fixation was performed on CAGE and L5-S1 vertebral body under fluoroscopy [14].

②OLIF: For L2-L5 levels, OLIF surgery is performed in accordance with standard procedure. The patient was placed in the right lateral position and X-rays were used to identify the degenerative segment. (Scoliosis does not affect the choice of surgical approach).4-6 cm skin incision was made 6–10 cm anterior to the mid-portion of the marked disc (L2–4, two Incisions; L3–5 or L4–5, one Incision). The surgeon approached the retroperitoneal space by blunt dissection and anterior displacement of the peritoneum to expose psoas. After the discectomy, endplate cartilage was removed and inserted an intervertebral cage filled with homogeneous bone.

③PPSF: PPSP was performed in the last step, with anterior fixation of L5 vertebrae, L5 vertebrae are usually not screwed in the absence of significant osteoporosis. For the L2-S1 levels, PPSF was often performed on L2, L4 and S1; For the L3-S1 levels, PPSF was often performed on L3, L4 and S1; For the L4-S1 levels, PPSF was often performed on L4 and S1.

### Perioperative management

On the first day and third day after the operation, blood routine examination was repeated. Methylprednisolone, omeprazole, parecoxib, and rivaroxaban were used for conventional treatment 3 days after operation. Lumbar spine x-rays are repeated 1–2 days after surgery, with the duration postoperative activity determined by the patient’s status. Most patients were able to walk after surgery day 1–3, protected by a lumbar brace.

### Data collection

A total of 100 cases were included between November 2020 and April 2022. Demographics (sex, age, height, weight), body mass index (BMI) were converted to qualitative data at a cutoff of 24, laboratory data (preoperative Hematocrit (HCT), postoperative HCT), surgical data (operation duration, Surgical segments, intraoperative bleeding, blood transfusion.), and radiographic data, Hypertension, Diabetes were collected.

### Data calculation

**Fig. 1:**
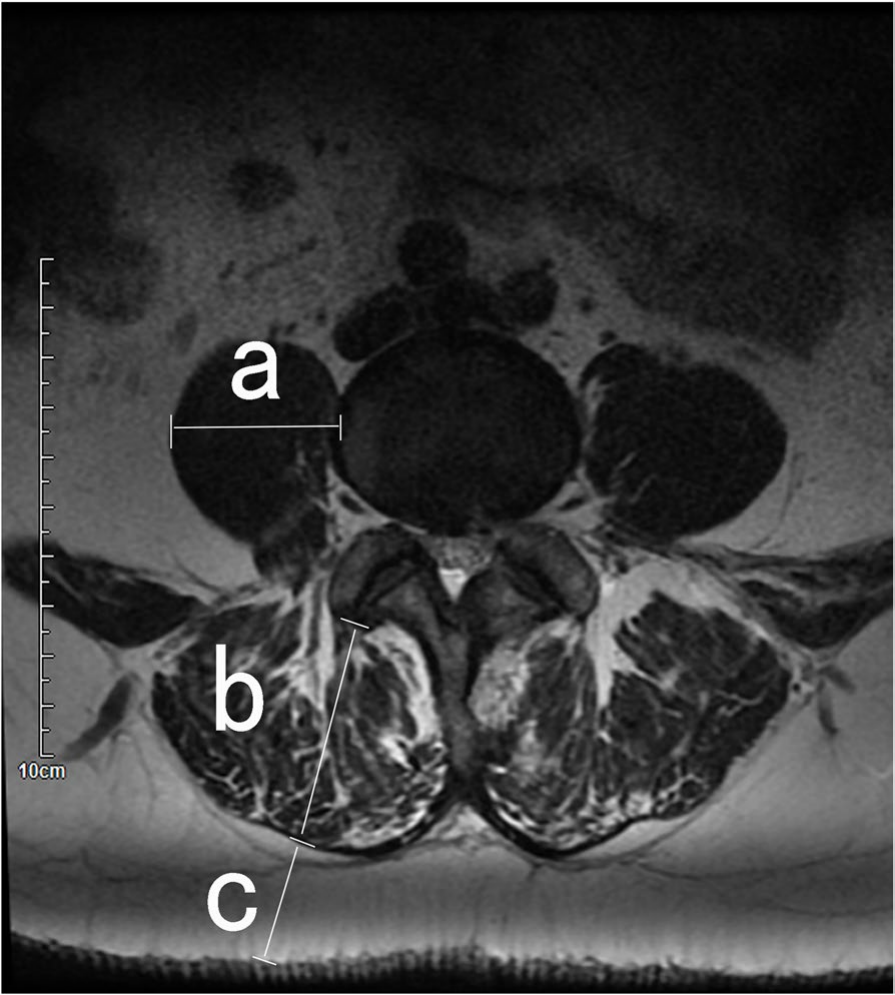
Diagram of the method used to measure the psoas (a) dorsal extensor group (b) and subcutaneous fat (c) at the leval of L4 using axis views was determined on T2-weighted MRI

Preoperative magnetic resonance imaging (MRI) was used to determine thickness of the psoas, thickness of the dorsal extensor group, and thickness of the subcutaneous fat. These measurements were made at the L4 level using image J (Fig. 1).

Calculation of hidden blood loss:①according to the formula of Nadler: PBV = k1 × height(m)3 + k2 × weight (kg) + k3 (for male: k1 = 0.3669, k2 = 0.03219, and k3 = 0.6041; for female: k1 = 0.3561, k2 = 0.03308, and k3 = 0.1833).②according to the Gross formula: total blood loss (TBL) = PBV (Hct_pre_ − Hct_post_)/Hct_ave_, where Hct_pre_ is the preoperative Hct, Hct_post_ is the second or third postoperative Hct, and Hct_ave_ is the average of Hct_pre_ and the Hct_post_.③according to the formula of Sehat: HBL = TBL - visible blood loss (VBL). Without post-operative drainage, the VBL is approximately equal to the intraoperative bleeding.

### Data analysis

The SPSS 25.0 software was used to analyze the data. The statistical results were described, with continuous variables shown as means and standard deviations, and classification shown as a percentage. To compare the differences between surgical segments, the ANOVA test has been used. Age, BMI, duration of surgery, diabetes, hypertension, psoas thickness, and dorsal extensor group thickness were identified as risk factors for HBL using Spearman’s correlation analysis and multivariate linear regression analysis. *P* < 0.05 was regarded as statistically significant.

## Results

### General information

To summarize all data, a total of 100 patients (37 males; average age was 65.41 ± 8,66 years) were reviewed retrospectively. And distinguish them based on quantitative or qualitative data, with quantitative data shown as means standard deviation. The patient demographic and clinical data (including blood loss results) are summarized in Tables 1 and 2.The hidden blood loss averaged 57% of the TBL. The average thickness of the dorsal extensor group was 38.40 ± 6.93 mm and the thickness of the subcutaneous fat was 17.62 ± 7.62 mm, which was similar to the study by Zhou et al.

**Table 1:** Patient demographic and clinical information (Qualitative data)

Variable	Classify	Quantity (Proportion)
Sex	male	37(37%)
	female	63(63%)
BMI	<=24	29(29%)
	> 24	71(71%)
Fusion segment	2	42(42%)
	3	46(46%)
	4	12(12%)
Hypertension	1^a^	29(29%)
	2^a^	71(70%)
Diabetes	1^a^	17(17%)
	2^a^	83(83%)

1^a^with hypertention or diabetes; 2^a^without hypertention or diabetes

**Table 2:** Patient demographic and clinical information (Quantitative data)

Variable	Mean ± SD	95% CI
Age(years)	65.41 ± 8.66	63.41–67.13
Operation duration(h)	6.36 ± 1.08	6.15–6.58
Preoperative Hct	42.58 ± 3.78	41.82–43.29
Postoperative Hct	36.70 ± 4.84	35.74–37.66
Thickness of the dorsal extensor group (mm)	38.40 ± 6.93	37.03–39.78
Thickness of the subcutaneous fat (mm)	17.62 ± 7.62	16.11–19.14
Thickness of the psoas (mm)	30.76 ± 7.04	29.36–32.16
TBL(ml)	645.59 ± 376.37	570.91–720.27
HBL(ml)	421.39 ± 337.45	354.43–488.35
Proportion OF HBL (%)	57 ± 26	53–62

### Diabetes and fusion number

Post-operative Hct change and hidden blood loss were higher in diabetes patients than in non-diabetic patients (t-test Hct change: 6.37 ± 3.23 VS 3.33 ± 1.60,*P* < 0.001;HBL: 465.61 ± 337.54 ml VS 205.51 ± 247.08 ml, *P* = 0.001) (Table 3).ANOVA tests were performed to identify independent factors associated with total blood loss and the number of surgical levels. The fusion number did not have a significant effect on the HBL or TBL(F = 1.450,*p* = 0.240),as shown in Table 4.

**Table 3:** Comparison of HBL between with diabetes and without diabetes

	Preoperative Hct	Postoperative Hct	Hct changes	HBL (ml)
1^a^(17%)	42.83 ± 3.27	36.45 ± 4.63	6.37 ± 3.23	465.61 ± 337.54
2^a^(83%)	41.21 ± 5.56	37.87 ± 5.74	3.33 ± 1.60	205.51 ± 247.08
t	1.167	−1.104	5.786	3.692
p	0.258	0.272	0.000	0.001

1^a^with diabetes; 2^a^without diabetes

**Table 4:** Comparison of HBL between fusion segments

Fusion segments(Proportion)	HBL(ml)	95%CI
2(42%)	360.46	275.39–445.53
3(46%)	449.30	332.94–565.64
4(12%)	527.68	334.75–717.61
F	1.450	
p	0.240	

### Spearman relative analysis and multi-factor linear regression

As shown in Table 5, according to the professional knowledge, we analyzed the independence of each variable and preliminarily screened for 12 variables (Age, Sex, BMI, Diabetes, Hypertension, Operation duration, Surgical segments, Preoperative HCT, Postoperative HCT, thickness of the psoas, thickness of the dorsal extensor group, and thickness of the subcutaneous fat).

**Table 5:** Spearman relative analysis

Variable	*P* value
Age	0.179
Sex	0.589
BMI	0.634
Hypertention	0.034
Diabetes	0.003
Fusion segment	0.090
Operation duration	0.010
Preoperative Hct	0.996
Postoperative Hct	0.000
Thickness of the psoas	0.743
Thickness of the dorsal extensor group	0.144
thickness of the subcutaneous fat	0.296

Based on the result of Spearman relative analysis combined with clinical analysis, 12 variables were included in the Multi-factor linear regression (Table 6). We identified several potential risk factors for postoperative hidden blood loss using multi-factor regression, including diabetes mellitus (*p* < 0.05), hypertension (p < 0.05), postoperative HCT (p < 0.05), psoas thickness (p < 0.05), and thickness of the dorsal extensor group (p < 0.05). Psoas thickness and dorsal extensor group thickness were favorably correlated with HBL. However, no link was found between age, BMI, fusion segment, operation duration, and subcutaneous fat thickness.

**Table 6:** Results of multi-factor line regression method for HBL

Variable	Unstandardized β	Standardized β	t	P
age	3.344	0.086	1.069	0.288
BMI	− 118.216	0.644	−0.160	0.450
diabetes	− 264.662	−3.883	− 0.296	0.000*
hypertention	183.656	0.248	3.145	**0.002**
Fusion segment	25.422	0.051	0.580	0.563
Operation duration	20.961	0.073	0.705	0. 483
Postoperative Hct	−44.925	−0.614	−7.118	0.000*
subcutaneous fat^a^	−3.473	−0.078	−0.973	0.334
dorsal extensor group^a^	9.052	0.186	2.081	**0.040**
psoas^a^	12.750	0.266	3.145	**0.002**

*HBL* hidden blood loss, a the thickness of the Variable *p < 0.001

## Discussion

While minimally invasive spine surgery has had some positive results, the hidden blood loss was also noticeable. In our study, the mean total blood loss was 645.59 ± 376.37 ml and the mean hidden blood loss was 421.39 ± 337.45 ml (representing 57 ± 26% of TBL). To further detect some differences in HBL between the different groups, we have compared this study with previous studies (see Table 7). Upon review of the literature, we found that hidden blood loss in ALIF,OLIF,XLIF,MIS-TLIF and PLIF were 400.5 ± 207.7 ml, 797 ± 275 ml, 258 ± 168 mL, 488.4 ± 294.0 ml and 449 ± 191 mL,respectively [7, 11, 12, 15, 16]. Fei Lei [15] et al. indicated that although the Wiltse approach better exposes the site of the operation and reduces the separation of the paraspinal muscles, bleeding on the surface of the bone after osteotomy and from the rupture of the spinal venous plexus is unavoidable.

**Table 7:** Hidden blood loss in different lumbar fusion procedures

author	surgery	levels	VBL	HBL
The present study	ALIF+OLIF	2.7 ± 0.7 levels	224.2 ± 156.0 mL	421.4 ± 337.5 ml
Yuichiro Mima	XLIF	2.5 ± 0.6 levels	33 ± 52 mL	258 ± 168 mL
H. Ju	ALIF	2.5 ± 1.1 levels	700.1 ± 562.3 mL	400.5 ± 207.7 ml
Koichiro Shima	OLIF	2.5 ± 1.0 levels	122 ± 118 ml	797 ± 275 ml
Yuanxing Zhou	MIS-TLIF	1.4 ± 0.6 levels	284.2 ± 108.4 ml	488.4 ± 294.0 ml
Fei Lei	PLIF	1.5 ± 0.6 levels	593 ± 286 ml	449 ± 191 mL

*HBL* hidden blood loss, *VBL* visible blood loss

As shown in Table 7, the mean segment of the present study was close to that of Mima, Ju, and Shima et al. However, hidden blood loss and visible bleeding showed significant differences. We can draw the interesting conclusion that the hidden blood loss in our study lies between OLIF and ALIF alone, and the same for the visible blood loss. It is worth noting that Zhu et al. reported a hidden blood loss of 809 ml for a single-segment OLIF procedure [17]. On this basis we can speculate that OLIF plays an important role in the minimally invasive hybrid approach with hidden blood loss [11, 12]. The total amount of blood lost during minimally invasive surgery was lower than during open surgery, but the percentage of hidden blood loss was higher. We suggest that this is related to postoperative ALIF and OLIF (residual inter-tissue blood leakage between tissues) without drainage, and the peritoneum absorption of some of the bleeding [11].

Hypertension(*p* = 0.002) and Diabetes(*p* < 0.001) were a positive influencing factor of hidden blood loss, which were contrary to the findings of Man Hu et al. [18]. The author believes that this discrepancy may stem from the surgical approach (Percutaneous endoscopic transforaminal discectomy (PETD) for lumbar disc herniation no bone tissue damage and minimal soft tissue damage). Kara also showed a significant correlation between mean arterial pressure (MAP) preoperatively and intraoperative bleeding [19]. Abnormal perioperative blood glucose (including hyperglycaemia, hypoglycaemia and fluctuations in blood glucose) increased mortality in surgical patients and increased the incidence of complications such as infection, non-healing wounds and cardiovascular and cerebrovascular events, which was also a risk factor for postoperative hidden blood loss. Effective perioperative management of hypertension and diabetes reduces complications, mortality and total hospital costs [20, 21].

Our study considered that thickness of the psoas and thickness of the dorsal extensor group were the key factor of HBL in minimally invasive hybrid approach. However, thickness of the subcutaneous fat did not turn out to be a risk factor of HBL. Similar results to Kara’s, but lumbosacral maximum subcutaneous fat thickness (LSMSF) was a risk factor in his study [19]. Our measurement methods are inconsistent but the results are similar to Zhou [16] et al., and yet their multi-factor regression did not include hypertension and diabetes mellitus.

Operation duration was illuminated in our study, which was not a positive influencing factor of hidden blood loss (*p* = 0. 483). In contrast, Cai found that time to operation was an independent risk factor in elderly patients undergoing ACDF [22]. The mean operation duration was 6.36 ± 1.08 h, which was significantly higher than other studies [3, 10, 16, 17]. The reasons are as follows: the minimally invasive hybrid approach consists of three procedures, in other words, three different positions. Each transposition requires repositioning and disinfection, so it takes a long time to prepare for surgery.

By segmental comparison (360.46 ml for 2 segment surgery, 449.30 ml for 3 segment surgery and 527.68 ml for 4 segment surgery), we found that the amount of hidden blood loss increased with the increase in segment, but the difference between the groups was not statistically significant (*p* > 0.05), which is consistent with the outcome of the research by Yoji Ogura et al. [6]. Xu also demonstrated that in PLIF surgery, surgical segments did not affect the percentage of postoperative hidden blood loss [23]. Nevertheless, in adolescent scoliosis surgery, the fused segment was often an important risk factor [24].

How to reduce the incidence of hidden blood loss effectively, The author believes that two points should be emphasized, namely reasonable surgical strategy and hemostatic drugs. Yin [25] suggested that reducing intraoperative bleeding can reduce the incidence of recessive blood loss. This requires the operators to be skilled in anatomy and surgical techniques to avoid unnecessary intraoperative trauma. Wen [26] et al. found that skilled management shortened operative time and reduced hidden blood loss.

Tranexamic acid, a frequently used haemostatic drug in orthopedic surgery, has been widely employed in spinal surgery to reduce intraoperative bleeding and hidden blood loss with the absence of thrombo-embolic events [27, 28]. The current study focuses on the use of tranexamic acid in posterior spinal fusion, mainly by intravenous drip and topical application; Zhinan Ren [29] applied a topical tranexamic acid dip (1 g tranexamic acid in 100 mL saline solution) for 5 minutes before wound closure and showed a significant reduction in postoperative invisible blood loss, similar to the findings of Shi [30] et al. On the other hand, Zhu [31] advocated one intravenous dose of tranexamic acid 30 min before surgery and one dose 3 h after the start of surgery, while Zheng [32] suggested a combination of intravenous drip and topical application to reduce intraoperative bleeding. This study also showed that tranexamic acid does not increase the risk of thrombosis and does not have a serious impact on the patient’s liver or kidney function. As mentioned above, satisfactory results were obtained with both modalities, but further trials are needed to determine their advantages and disadvantages [33]. A multifunctional cocktail, also applied topically, was studied by Jiang to reduce postoperative bleeding and to reduce pain in patients. Unlike the topical application of tranexamic acid, a multifunctional cocktail was applied after closure of the posterior lumbar surgical incision [34].

Our understanding of treatment using a minimally invasive hybrid approach:①Studies have previously shown that the L4–5 segment is an independent risk factor for hidden blood loss in ALIF surgery, which greatly increases the amount of hidden blood loss and the difficulty of the procedure [11].②In contrast, OLIF surgery for the L5-S1 segment is difficult to perform due to iliac crest obstruction [4].③Whether only ALIF approach or only OLIF approach, there are many difficulties in multiple segments of lumbar degenerative disease (especially including L5-S1 level).④The minimally invasive hybrid surgery is the embodiment of the minimally invasive concept. It combines the advantages of various minimally invasive procedures to achieve optimal results and minimize injury. Therefore, we performed a minimally invasive combination approach for multiple lumbar degenerative disease, ALIF for L5-S1 segment and OLIF for L2-L5, effectively combining the advantages of both approaches with significant results.

Based on our experience, and in combination with our findings, we identified possible risk factors for postoperative hidden blood loss in patients.① the patient with diabetes or hypertension ②the patient with greater thickness of the psoas major and dorsal extensor group muscles③ postoperative application of anticoagulants ④ postoperative early movement out of bed ⑤difficulty in Haemostasis (Small incision with inadequate exposure, especially in PPSF) ⑥ the lack of drainage ⑦Bleeding from other sources, such as from the gastrointestinal tract. Above are factors that may cause hidden blood loss, and further studies are necessary to verify these.

### Study limitations

Our study has limitations, such as the small amount of patient data collected and the need for a larger randomized controlled trial to further validate the trial. Additionally,measuring the thickness of the patient’s psoas major muscle and the dorsal extensor group using MRI axial slices, while measured by three different observers, inevitably results in systemic errors in manual measurements.

## Conclusions

With the development of spinal minimally invasive techniques, the amount of visible blood loss is gradually decreasing, but the existence of hidden blood loss cannot be ignored. In our study, the amount of hidden blood loss in the minimally invasive hybrid approach was 421.39 ± 337.45 ml, representing 57 ± 26% of total blood loss. The management of perioperative fluids should not be neglected, especially in patients with diabetes, hypertension and preoperative MRI revealing greater thickness of the psoas major and dorsal extensor muscles, which are often associated with greater hidden blood loss. For surgeons, a reasonable preoperative assessment of these risk factors is essential for the patient’s rapid postoperative recovery and reduction of the risk of adverse events.

## Data Availability

Data cannot be provided due to identifying information of participants but are available from the corresponding author on reasonable request.

## Abbreviations

LIF: lumbar interbody fusion
LDD: lumbar degenerative diseases
ALIF: anterior lumbar interbody fusion
OLIF: oblique lateral lumbar interbody fusion
XLIF: extreme lateral interbody fusion
LLIF: lateral lumbar interbody fusion
HBL: hidden blood loss
PPSF: percutaneous pedicle screw fixation
BMI: body mass index
HCT: Hematocrit
MRI: magnetic resonance imaging
PBV: preoperative blood volume
TBL: total blood loss
VBL: visible blood loss
MIS-TLIF: Minimal Invasive Surgery-Transforaminal Lumbar Interbody Fusion
PLIF: posterior lumbar interbody fusion
PETD: Percutaneous endoscopic transforaminal discectomy
MAP: mean arterial pressure
ACDF: anterior cervical decompression and fusion
